# Cortical microstructural correlates of astrocytosis in autosomal-dominant Alzheimer disease

**DOI:** 10.1212/WNL.0000000000009405

**Published:** 2020-05-12

**Authors:** Eduard Vilaplana, Elena Rodriguez-Vieitez, Daniel Ferreira, Victor Montal, Ove Almkvist, Anders Wall, Alberto Lleó, Eric Westman, Caroline Graff, Juan Fortea, Agneta Nordberg

**Affiliations:** From the Memory Unit, Department of Neurology (E.V., V.M., A.L., J.F.), Hospital de la Santa Creu i Sant Pau, Biomedical Research Institute Sant Pau, Universitat Autònoma de Barcelona; Centro de Investigación Biomédica en Red de Enfermedades Neurodegenerativas, CIBERNED (E.V., V.M., A.L., J.F.), Madrid, Spain; Department of Neurobiology (E.R.-V., D.F., O.A., E.W., A.N.), Care Sciences and Society, Center for Alzheimer Research, Division of Clinical Geriatrics, and Division of Neurogeriatrics (C.G.), Karolinska Institutet, Stockholm Department of Psychology (O.A.), Stockholm University; The Aging Brain Unit (O.A., A.N.) and Unit for Hereditary Dementias (C.G.), Theme Aging, Karolinska University Hospital, Stockholm; Department of Surgical Sciences, Section of Nuclear Medicine & PET (A.W.), Uppsala University, Sweden; and Department of Neuroimaging (E.W.), Centre for Neuroimaging Sciences, Institute of Psychiatry, Psychology and Neuroscience, King's College London, United Kingdom.

## Abstract

**Objective:**

To study the macrostructural and microstructural MRI correlates of brain astrocytosis, measured with ^11^C-deuterium-L-deprenyl (^11^C-DED)–PET, in familial autosomal-dominant Alzheimer disease (ADAD).

**Methods:**

The total sample (n = 31) comprised ADAD mutation carriers (n = 10 presymptomatic, 39.2 ± 10.6 years old; n = 3 symptomatic, 55.5 ± 2.0 years old) and noncarriers (n = 18, 44.0 ± 13.7 years old) belonging to families with mutations in either the presenilin-1 or amyloid precursor protein genes. All participants underwent structural and diffusion MRI and neuropsychological assessment, and 20 participants (6 presymptomatic and 3 symptomatic mutation carriers and 11 noncarriers) also underwent ^11^C-DED-PET.

**Results:**

Vertex-wise interaction analyses revealed a differential relationship between carriers and noncarriers in the association between ^11^C-DED binding and estimated years to onset (EYO) and between cortical mean diffusivity (MD) and EYO. These differences were due to higher ^11^C-DED binding in presymptomatic carriers, with lower binding in symptomatic carriers compared to noncarriers, and to lower cortical MD in presymptomatic carriers, with higher MD in symptomatic carriers compared to noncarriers. Using a vertex-wise local correlation approach, ^11^C-DED binding was negatively correlated with cortical MD and positively correlated with cortical thickness.

**Conclusions:**

Our proof-of-concept study is the first to show that microstructural and macrostructural changes can reflect underlying neuroinflammatory mechanisms in early stages of Alzheimer disease (AD). The findings support a role for neuroinflammation in AD pathogenesis, with potential implications for the correct interpretation of neuroimaging biomarkers as surrogate endpoints in clinical trials.

Alzheimer disease (AD) is a complex disorder in which multiple pathophysiologic features coexist.^1^ In a small proportion of patients, AD is hereditary due to autosomal-dominant mutations^2^ with an early and rather predictable mutation-specific age at onset, allowing the investigation of presymptomatic brain changes.^2,3^

Neuroinflammation is postulated as a key player in AD pathogenesis.^4,5^ While most PET imaging studies of neuroinflammation have studied microgliosis,^6,7^ few PET tracers exist for astrocytosis. The most common is ^11^C-deuterium-L-deprenyl (^11^C-DED), which targets monoamine oxidase-B (MAO-B).^8–11^ Using ^11^C-DED-PET in a longitudinal autosomal-dominant AD (ADAD) cohort, we previously reported presymptomatic astrocyte activation followed by decline along disease progression.^12^

The complexity of AD requires multimodal approaches. Using structural and diffusion MRI, we recently proposed a model of gray matter (GM) changes in sporadic AD,^13–16^ in which an early presymptomatic phase of decreased cortical mean diffusivity (MD) and increased cortical thickness (CTh) is followed by increased cortical MD and decreased CTh at symptomatic stages. Although the origin of these structural changes is unclear, previous biological evidence suggests a role for neuronal or glial remodeling and hypertrophy.^15,17,18^ Whether astrocytosis has a measurable structural correlate in AD is unknown. Identifying structural correlates of brain inflammation is of potential utility for clinical trial design, specifically when interpreting the structural changes observed in immunization or in trials targeting neuroinflammation. In this proof-of-concept study, we aimed to (1) assess astrocytosis in ADAD using ^11^C-DED-PET; (2) investigate microstructural and macrostructural measures in ADAD; and (3) investigate the microstructural and macrostructural correlates of astrocytosis in ADAD.

## Methods

### Study design and participants

Individuals from families with known ADAD mutations were recruited through the Unit for Hereditary Dementias, which provides genetic counseling at Theme Aging, Karolinska University Hospital (Stockholm, Sweden). The participants with ADAD in this study are part of an ongoing prospective research study at Karolinska Institutet that started in 1993 involving families that carry 1 of 4 mutation types. All family members were invited to participate, and those who accepted were included. Recruitment was performed blind to participants' mutation status. Therefore, the study includes mutation carriers and noncarriers, all recruited and examined following identical procedures, without known selection bias.

Symptom onset in mutation carriers is defined as the time at which the first clinically relevant cognitive symptoms appeared, as either experienced by the patient or noticed by near relatives. In this cohort, the average age at onset was earliest (36 ± 2 years) in *PSEN1* Ile143Thr mutation carriers, while it was similar in carriers of the other 3 mutations: *PSEN1* His163Tyr (52 ± 7 years), *APP*swe KM670/671NL (54 ± 5 years), and *APP*arc Glu693Gly (56 ± 3 years).^19^ The average age at onset in each family was calculated from medical records for disease onset in individuals from that family (5, 9, 24, and 12 individuals for the 4 mutation types, respectively). The estimated number of years to symptom onset (EYO) was calculated for each carrier or noncarrier participant by subtracting the individual's age from the average age at onset for the respective family. The concept of EYO in noncarriers is artificial. We only use it in the interaction analyses to have the participants in the same temporal dimension.

In our study, symptomatic carriers had been clinically diagnosed with either mild cognitive impairment^20^ or AD dementia.^21^ Presymptomatic carriers had no cognitive complaints and did not fulfill the criteria for mild cognitive impairment or AD dementia. Clinicians and researchers in contact with or examining the ADAD research participants were blind to the mutation status. Diagnoses were made during a consensus meeting where a geriatrician/neurologist, a neuropsychologist, and a nurse discussed the outcome of the participant assessment.

The study included 31 participants (table 1). All available ADAD mutation carriers who had both MRI and diffusion-weighted imaging (DWI) data were selected. An age- and sex-matched (to both presymptomatic and symptomatic carriers) group of noncarriers who also had MRI and DWI data were used as a control group for the mutation carriers. All participants underwent a comprehensive clinical and imaging examination that included a medical history, neurologic and psychiatric examination, EEG, MRI, *APOE* genotyping, and neuropsychological assessment. Moreover, a subset of participants (n = 20, table 2) underwent a ^11^C-DED PET scan. This was acquired within 3.8 ± 3.7 months of the MRI scan, except for 2 participants: 1 presymptomatic carrier had the ^11^C-DED PET scan 1.7 years before the MRI and 1 symptomatic carrier had the scan 3.3 years after the MRI. When analyses involving comparisons between MRI and PET imaging data were repeated excluding these 2 participants, the results did not change significantly (not shown).

**Table 1:**
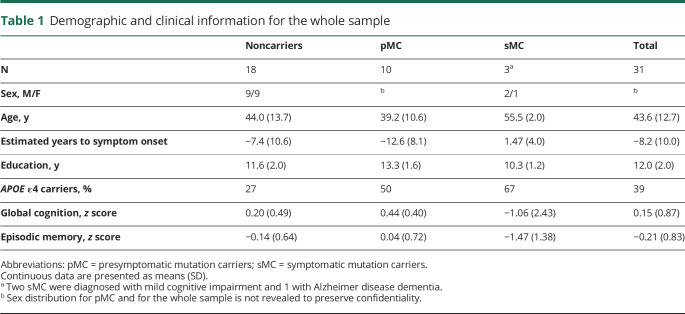
Demographic and clinical information for the whole sample

**Table 2:**
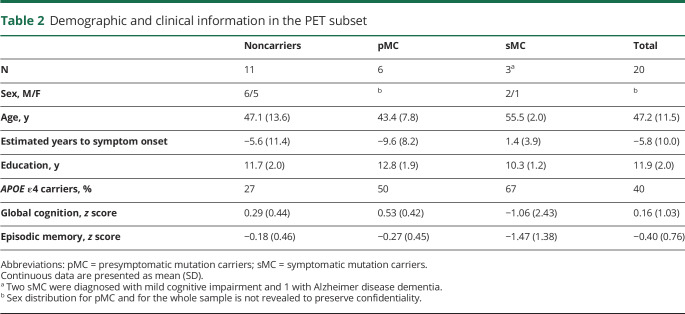
Demographic and clinical information in the PET subset

### Standard protocol approvals, registrations, and patient consents

All participants provided written informed consent to participate in the study, which was conducted according to the Declaration of Helsinki and subsequent revisions. Ethical approval was obtained from the regional Human Ethics Committee of Stockholm and the Faculty of Medicine and Radiation Hazard Ethics Committee of Uppsala University Hospital, Sweden.

### MRI acquisition and processing

All participants (n = 31) underwent a structural 3D T1 magnetization-prepared rapid acquisition gradient echo sequence and a diffusion tensor imaging (DTI) MRI sequence in a 3T Siemens (Munich, Germany) Trio scanner. The acquisition measures of T1 MRI included the following: repetition time/echo time (TR/TE) 1,780/3.42 ms, inversion time 900 ms, 192 sagittal slices, voxel size 1 × 1 × 1 mm^3^, and flip angle = 9°. DTI was performed using a spin echoplanar imaging sequence (TR/TE 8,000/97 ms, 60 axial slices, voxel size 2 ×2 ×2.4 mm^3^) with 30 orientations for the diffusion-sensitizing gradients (b-value of 1,000 s/mm^2^). Further details of the procedure can be found elsewhere.^12,22^

Structural MRI was preprocessed using FreeSurfer 6.0 (surfer.nmr.mgh.harvard.edu).^23^ All cortical segmentations were inspected visually to detect processing errors, which were corrected if necessary as is customary in MRI surface-based analyses.^14,15^ Of the initial 33 participants, 2 (1 mutation carrier and 1 noncarrier) had been excluded from the analysis because of severe segmentation errors (6%), thus 31 were finally included (table 1).

Diffusion imaging data were processed with an in-house surface-based DTI approach,^15^ which uses tools from the FSL (FMRIB Software Library) (fsl.fmrib.ox.ac.uk/fsl/fslwiki, version 5.0.9) and FreeSurfer 6.0 packages. This surface-based approach takes advantage of recent methodologic advances^24–26^ to overcome the limitations of traditional voxel-based approaches when used in analyses of the cortical mantle. First, it reduces the contribution from CSF and white matter signal in GM voxels that can confound the cortical MD measures. Second, it applies a surface-based smoothing procedure, as it has been shown that volume-based analysis techniques may be sensitive to the across-voxel smoothing kernel size.^27^ In the surface-based DTI approach, images were motion-corrected, skull-stripped, and diffusion tensor–fitted. The diffusion images were then coregistered to each participant's T1 native space using the *bbregister* tool in FreeSurfer 6.0. The cortical MD maps resulting from the DTI fitting were then sampled in the midpoint between white and pial surfaces generated by FreeSurfer, projected onto the participant's cortical surface space, and registered to the FreeSurfer standard space for subsequent analysis.

### PET image acquisition and processing

A subset of participants (n = 20) underwent ^11^C-DED PET imaging at the Uppsala PET Centre, Uppsala University, Sweden. Briefly, 60-minute dynamic ^11^C-DED images were acquired on ECAT EXACT HR+ (Siemens/CTI) and GE (Chicago, IL) Discovery ST PET/CT scanners (mean injected dose, 221 ± 65 MBq), reconstructed, and motion corrected.^12,22^ All PET emission data were reconstructed with filtered backprojection using a 4-mm Hanning filter, resulting in a transaxial spatial resolution of 5 mm in the field of view. The matrix included 128 × 128 pixels, and a zoom factor of 2.5 was used. All 19 reconstructed frames (4 × 30 s, 8 × 60 s, 4 ×300 s, and 3 ×600 s) were realigned for motion correction using the second frame as reference, with subsequent time frames being successively realigned to the previous one. For ^11^C-DED PET quantification, a modified-reference Patlak model^10,28^ was applied to the 20–60 minutes dynamic ^11^C-DED PET images using the cerebellar GM as modified reference region to generate individual parametric Patlak slope images (units: min^−1^), assuming a cerebellar GM slope of 0.01 min^−1^. ^11^C-DED binding was then expressed as the ratio of ^11^C-DED slope in each brain voxel to that in the cerebellar GM.

Once ^11^C-DED PET binding had been calculated, 10–60 minutes averaged PET images were used to coregister the PET volume to each participant’s native T1 using the *mri_coreg* tool in FreeSurfer 6.0. Although the parametric ^11^C-DED images were originally generated in native ^11^C-DED PET space, the images were subsequently projected onto the cortical surface space for direct comparison between the ^11^C-DED PET and MRI data. The cortical ^11^C-DED binding was sampled in the midpoint between pial and white matter FreeSurfer surfaces as in the diffusion analyses.

### Neuropsychological assessment

Participants were assessed using a comprehensive battery of neuropsychological tests, including memory, attention, language, executive, and visuospatial functions.^29^ Raw scores were converted to *z* scores using a reference group from the Karolinska University Hospital, and combined into 2 composite scores for global cognition (9 subtests) and episodic memory (3 subtests).^29^ Cronbach α was used to assess the internal consistency of each composite score. The episodic memory composite (Cronbach α = 0.73) was useful to capture early impairment,^29^ while the global cognitive composite (Cronbach α = 0.67) represented an aggregate of various nonmemory domains.^29^ The neuropsychological assessment was performed within 1.0 ± 3.3 months from the date of the MRI scan.

### Statistical analysis

Before any statistical data analysis of the PET and MRI, a 2D full-width half-maximum Gaussian kernel of 20 mm across the cortical mantle was applied to ^11^C-DED PET, MD, and CTh surfaces.

To address the main objective of this study, investigating the microstructural and macrostructural MRI correlates of astrocytosis using ^11^C-DED PET at different stages of ADAD, the statistical analysis was conducted in 3 steps.

In step 1, we assessed whether mutation carriers and noncarriers had different associations with EYO for 3 imaging modalities: ^11^C-DED PET, cortical MD, and CTh. To this end, vertex-wise linear regression models were assessed in FreeSurfer, with each imaging measure as a dependent variable and EYO as the independent predictor. We then statistically tested for a differential relationship of each imaging modality with EYO between mutation carriers (n = 9 for ^11^C-DED PET, n = 13 for cortical MD and CTh) and noncarriers (n = 11 for ^11^C-DED PET, n = 18 for cortical MD and CTh), using sex as covariate.

In step 2, to further investigate ^11^C-DED PET, cortical MD, and CTh differences across disease stages, the mutation carriers were stratified into presymptomatic or symptomatic, and these 2 subgroups were each compared to the noncarrier group. Vertex-wise general linear models were assessed in FreeSurfer with each imaging measure as a dependent variable, and age and sex as covariates. Group comparisons were carried out between each of the mutation carrier groups (n = 6 presymptomatic and n = 3 symptomatic for ^11^C-DED PET, n = 10 presymptomatic and n = 3 symptomatic for cortical MD and CTh) and the noncarrier group (n = 11 for ^11^C-DED PET, n = 18 for cortical MD and CTh).

In step 3, we investigated the local association between ^11^C-DED binding and cortical microstructural and macrostructural MRI measures using vertex-wise correlation analyses between ^11^C-DED binding and cortical MD, and between ^11^C-DED binding and CTh, in FreeSurfer. These associations were tested for the mutation carrier group alone (n = 9).

All the vertex-wise analyses described above were corrected for multiple comparisons within FreeSurfer by using a cluster extension criterion in a Monte Carlo simulation with 10,000 repeats, with the family-wise error (FWE) correction settled at *p* < 0.05. Only clusters that survived the multiple-comparisons correction are shown. For each analysis, all significant clusters were isolated, averaged, and plotted in scatterplots or box-and-whisker plots for illustrative purposes.

Group analyses for continuous nonimaging variables (demographic, clinical, and neuropsychological variables) were performed using analysis of variance with Tukey post hoc corrections or Kruskal-Wallis tests, as appropriate. The χ^2^ test was applied for categorical variables. Statistical analyses were performed using R statistical software (r-project.org).

### Data availability

Anonymized data will be shared by request from any qualified investigator for the sole purpose of replicating procedures and results presented in the report provided that data transfer is in agreement with EU legislation on the general data protection regulation.

## Results

Demographic and clinical data are summarized in table 1 for the whole cohort (n = 31) and in table 2 for the subset of participants with ^11^C-DED PET image data (n = 20). There were no significant differences in age, proportion of mutation carriers, or proportion of symptomatic carriers between the whole cohort and the PET subset. There were no significant differences in age between noncarriers and presymptomatic or symptomatic carriers in the whole sample (table 1) or in the PET subset (table 2). There were no significant differences in the proportion of *APOE* ε4 allele carriers between groups in the whole cohort and in the PET subset. Sex information for the presymptomatic carriers and the whole cohort, as well as individual family membership of the data points in the figures, are not revealed for confidentiality reasons.

### Neuropsychological profiles

In the whole cohort (table 1), the presymptomatic carrier group did not differ significantly in global cognition or episodic memory from the noncarrier group. Symptomatic carriers had the lowest scores for global cognition (*z* = −1.06 ± 2.43) and episodic memory (*z* = −1.47 ± 1.38) of all groups. They had significantly poorer episodic memory scores than noncarriers (Mann-Whitney *Z* = −2.11, *p* = 0.035); however, the difference in global cognition between symptomatic carriers and noncarriers was not statistically significant. Similarly, in the subset of participants with PET imaging data (table 2), presymptomatic carriers did not significantly differ in either global cognition or episodic memory from the noncarriers. Symptomatic carriers tended to have lower episodic memory scores than noncarriers (Mann-Whitney *Z* = −1.95, *p* = 0.052), while the respective comparison for global cognition did not reach statistical significance.

### ^11^C-DED binding in mutation carriers

**Figure 1:**
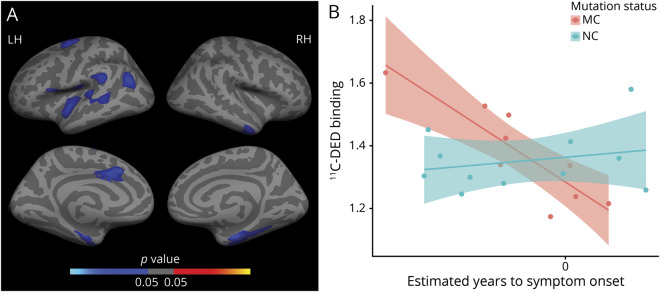
Differential relationship between estimated number of years to symptom onset (EYO) and ^11^C-deuterium-L-deprenyl (^11^C-DED) PET binding in mutation carriers (MC) vs noncarriers (NC) (A) Surface map represents clusters of significant differential relationship between ^11^C-DED PET binding and EYO depending on the mutation status (MC/NC). Only clusters surviving multiple-comparison correction are depicted (family-wise error [FWE]–corrected, *p* < 0.05); coordinates of all significant clusters are available in table e-1 (doi.org/10.5061/dryad.585581j). (B) Linear regression trajectories of ^11^C-DED PET binding averaged over the left hemisphere (LH) vs EYO in MC (red) and NC (blue), including 95% confidence bands around the model predictions (illustrative purposes). RH = right hemisphere.

**Figure 2:**
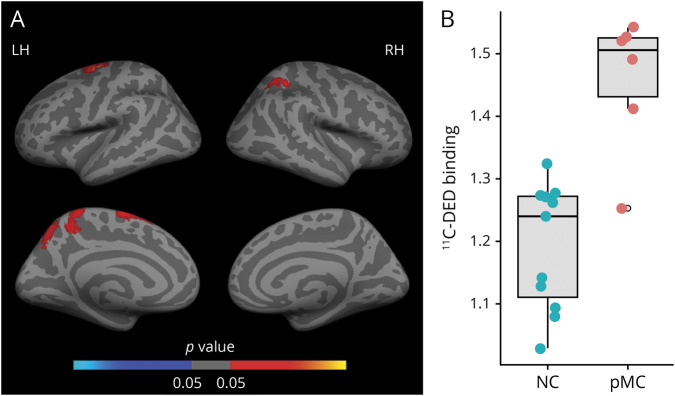
Group comparison of ^11^C-deuterium-L-deprenyl (^11^C-DED) PET binding in presymptomatic mutation carriers (pMC) vs noncarriers (NC) (A) Surface map represents clusters of increased (in red–yellow) ^11^C-DED PET binding in pMC compared to NC; only clusters surviving multiple-comparison correction are depicted (family-wise error [FWE]–corrected, *p* < 0.05); coordinates of all significant clusters are available in table e-2 (doi.org/10.5061/dryad.585581j). (B) Box-and-whisker plot compares the average ^11^C-DED binding over the left hemisphere (LH) between pMC (red) and NC (blue) (illustrative purposes). RH = right hemisphere.

Figure 1 shows the results of the linear regression models used to investigate the association between ^11^C-DED binding and EYO in mutation carriers and noncarriers. Figure 1A illustrates the clusters representing a significant differential relationship between ^11^C-DED binding and EYO depending on the mutation status (carrier/noncarrier) in the PET subset (n = 20). Significant clusters (FWE-corrected, *p* < 0.05) emerged mostly within bilateral temporal and frontal regions (figure 1A). For illustrative purposes, the average of all significant clusters was plotted separately for mutation carriers and noncarriers (figure 1B); coordinates of all significant clusters are available in table e-1 (doi.org/10.5061/dryad.585581j). ^11^C-DED binding was negatively associated with EYO in carriers while no significant association was observed in noncarriers. The vertex-wise whole brain group comparison of ^11^C-DED binding between presymptomatic carriers and noncarriers showed a pattern of increased ^11^C-DED binding in the presymptomatic carriers over clusters involving precentral, parietal, and precuneus regions (figure 2A; FWE-corrected, *p* < 0.05). For illustrative purposes, the comparison between presymptomatic carriers and noncarriers within the average of all significant clusters is shown by a box-and-whisker plot (figure 2B); coordinates of all significant clusters are available in table e-2 (doi.org/10.5061/dryad.585581j). In contrast, ^11^C-DED binding appeared to be similar or even decreased for the symptomatic carriers vs the noncarriers (figure e-1 and table e-3, doi.org/10.5061/dryad.585581j).

### Cortical microstructural and macrostructural measures in mutation carriers

**Figure 3:**
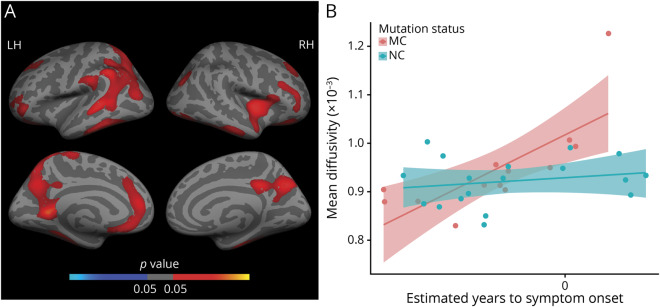
Differential relationship between estimated number of years to symptom onset (EYO) and cortical mean diffusivity (MD) in mutation carriers (MC) and noncarriers (NC) (A) Surface map represents clusters of significant differential relationship between cortical MD and EYO depending on the mutation status (MC/NC). Only clusters surviving multiple-comparison correction are depicted (family-wise error [FWE]–corrected, *p* < 0.05); coordinates of all significant clusters are available in table e-4 (doi.org/10.5061/dryad.585581j). (B) Linear regression trajectories of cortical MD averaged over the left hemisphere (LH) vs EYO in MC (red) and in NC (blue), including 95% confidence bands around the model predictions (illustrative purposes). RH = right hemisphere.

Figure 3 shows the results of the linear regression models used to investigate the association between cortical MD and EYO in mutation carriers and noncarriers. Figure 3A illustrates the clusters representing a significant differential relationship between MD and EYO depending on the mutation status (carrier/noncarrier) in the whole cohort (n = 31). The significant clusters (FWE-corrected, *p* < 0.05) in figure 3A show a widespread bilateral cortical pattern, including temporo-parietal, precuneus, posterior and anterior cingulate, and frontal regions. For illustrative purposes, the average of all significant clusters was plotted against EYO for mutation carriers and noncarriers separately (figure 3B). In mutation carriers, a pattern of increasing MD with disease progression (as measured by EYO) was observed, while no change in relation to EYO was seen in noncarriers. No result survived multiple comparisons when comparing the relationship of CTh with EYO between carriers and noncarriers (not shown).

**Figure 4:**
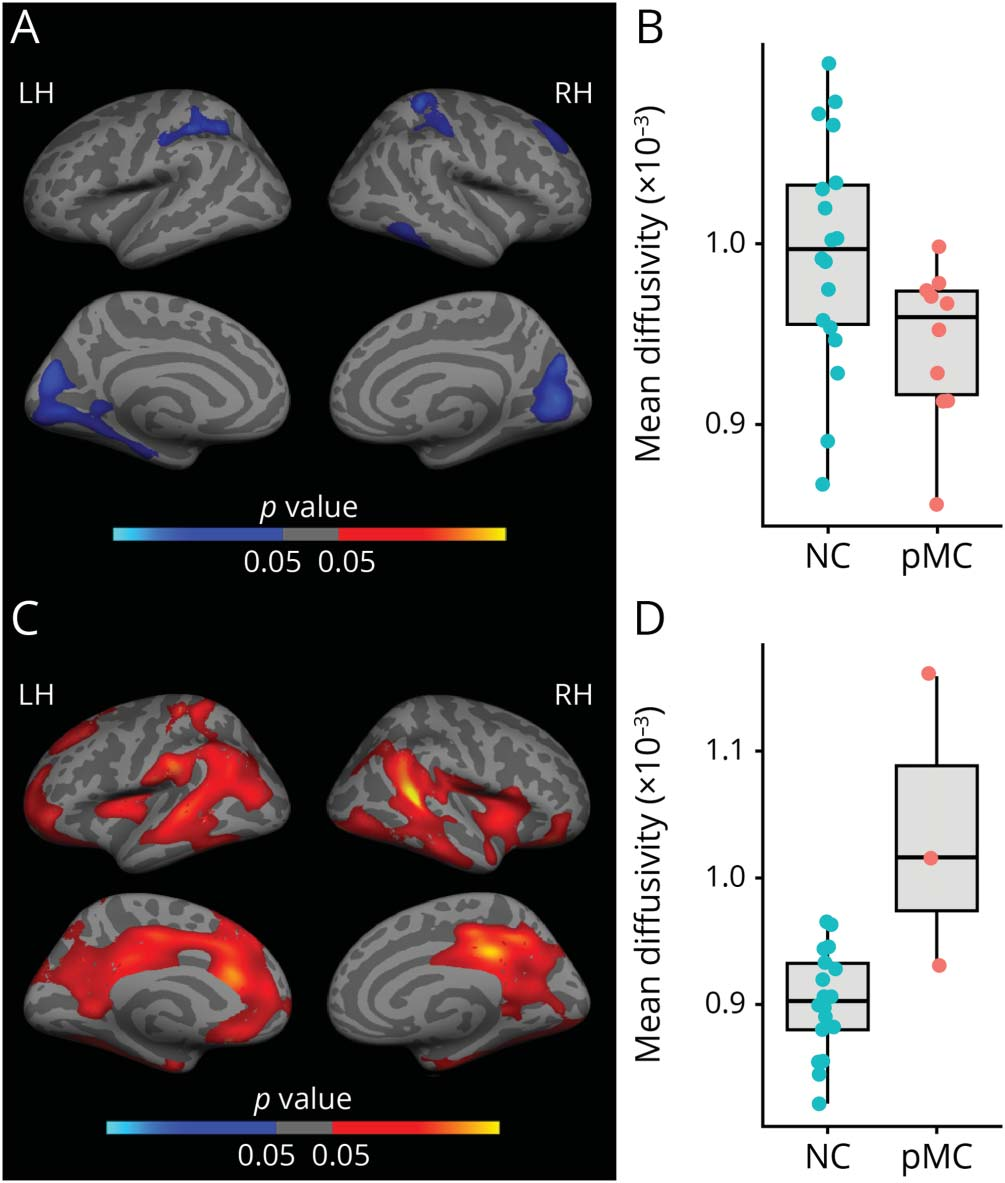
Group comparisons of cortical mean diffusivity (MD) in presymptomatic mutation carriers (pMC) and symptomatic mutation carriers (sMC) vs noncarriers (NC) (A) Surface map represents clusters of reduced (in blue) cortical MD in pMC compared to NC; only clusters surviving multiple-comparison correction are depicted (family-wise error [FWE]–corrected, *p* < 0.05); coordinates of all significant clusters are available in table e-5 (doi.org/10.5061/dryad.585581j). (B) Box-and-whisker plot compares the average cortical MD within the left hemisphere between pMC (red) and NC (blue) (illustrative purposes). (C) Surface map represents clusters of increased (in red) cortical MD in sMC compared to NC; only clusters surviving multiple-comparison correction are depicted (FWE-corrected, *p* < 0.05); coordinates of all significant clusters are available in table e-5. (D) Box-and-whisker plot compares the average MD within the left hemisphere (LH) between sMC (red) and NC (blue) (illustrative purposes). RH = right hemisphere.

Cortical MD was reduced in presymptomatic mutation carriers compared to noncarriers in clusters including parietal, frontal, temporal, and occipital regions (figure 4A). Figure 4B shows the average MD within all significant clusters in a box-and-whisker plot between presymptomatic carriers and noncarriers. On the other hand, cortical MD was increased over widespread clusters including bilateral temporo-parietal, cingulate, and frontal regions in symptomatic carriers vs noncarriers (figure 4C). Figure 4D shows the average MD within all significant clusters in a box-and-whisker plot between symptomatic carriers and noncarriers. Finally, there were subtle increases in CTh in presymptomatic carriers at the uncorrected level, and decreases in CTh in symptomatic carriers (figure e-2, doi.org/10.5061/dryad.585581j).

These analyses were repeated including *APOE* ε4 allele status (carrier vs noncarrier) as a covariate and the results did not change.

### Microstructural and macrostructural MRI correlates of ^11^C-DED binding

**Figure 5:**
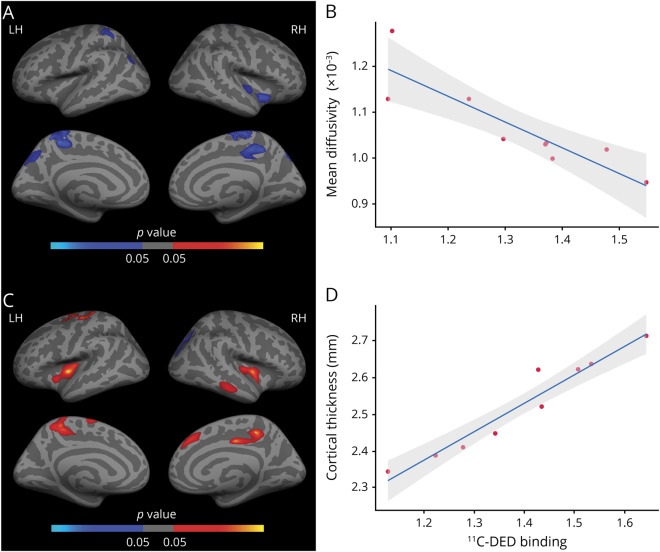
Local associations between ^11^C-deuterium-L-deprenyl (^11^C-DED) PET binding and cortical microstructure and macrostructure in mutation carriers (A) Surface map represents clusters of significant vertex-wise negative correlations (in blue) between astrocytosis as measured by ^11^C-DED PET binding and cortical mean diffusivity (MD). Only clusters surviving multiple-comparison correction are depicted (family-wise error [FWE]–corrected, *p* < 0.05); coordinates of all significant clusters are available in table e-6 (doi.org/10.5061/dryad.585581j). (B) Linear regression of cortical MD (vertical axis) vs ^11^C-DED PET binding (horizontal axis) averaged within the left hemisphere (LH), including a 95% confidence band around the model prediction (illustrative purposes). (C) Surface map represents clusters of significant vertex-wise correlations between astrocytosis as measured by ^11^C-DED PET binding and cortical thickness (CTh). Only clusters surviving multiple-comparison correction are depicted (FWE-corrected, *p* < 0.05); coordinates of all significant clusters are available in table e-6. (D) Linear regression of CTh (vertical axis) vs ^11^C-DED PET binding (horizontal axis) evaluated within the LH, including a 95% confidence band around the model prediction (illustrative purposes). RH = right hemisphere.

A vertex-wise map-to-map correlation analysis was carried out to assess the local association between ^11^C-DED binding and brain microstructure and macrostructure within the mutation carriers who had data on all imaging biomarkers (n = 9) (figure 5). Higher levels of ^11^C-DED binding were related to lower cortical MD in temporo-parietal regions (figure 5A, FWE-corrected, *p* < 0.05) and to increased CTh (figure 5C). The average of all significant clusters illustrating these significant associations between ^11^C-DED binding and cortical MD and CTh is plotted in figure 5, B and D, respectively.

## Discussion

In this study, we investigated the structural MRI correlates of brain astrocytosis as measured by ^11^C-DED PET in patients in early presymptomatic stages of ADAD. We report that ^11^C-DED binding has a measurable structural correlate in the form of decreased cortical MD and increased CTh in ADAD.

We first assessed ^11^C-DED binding in ADAD. Our results, using a surface-based approach, confirmed previously published results in the same cohort using regional and voxel-wise approaches^12^ in which ^11^C-DED binding peaked in early presymptomatic ADAD and then decreased with EYO toward the symptomatic stage. This result suggests that ^11^C-DED PET can track the inflammatory processes that occur in the early phases of the disease and highlights the role of neuroinflammation in AD pathogenesis. Preclinical studies have shown that astrocytes contribute to the clearance of β-amyloid species.^30,31^ The exposure of astrocytes to soluble β-amyloid species was reported to promote astrocytic MAO-B upregulation,^32^ which may explain the observed high ^11^C-DED binding in presymptomatic ADAD. In contrast, at late disease stages there is preclinical evidence for astrocytic dysfunction and atrophy observed in aged transgenic mouse brains.^17^ In our study, the observed decline in ^11^C-DED binding toward the symptomatic stage may thus be a sign of astrocyte cell loss or of progressive changes in the astrocyte phenotype indicating loss of function. Importantly, similarly to previous works,^12^ we observed a diffuse pattern of increased ^11^C-DED binding involving parietal and precuneus areas. These areas are susceptible to amyloid deposition but are relatively spared of tau deposition at early disease stages. Our results thus suggest that increased CTh (and reduced MD) are related to inflammation in early preclinical stages whereas reduced CTh (and increased MD) are more closely related to tau aggregation, which drives local neurodegeneration in later preclinical and prodromal AD stages.^33^

Only 2 previous studies have reported cortical MD decreases in presymptomatic ADAD. Fortea and collaborators^34^ found decreased cortical MD in a group of presymptomatic *PSEN1* carriers. These findings were later replicated in a separate cohort reported by Ryan and collaborators,^35^ who found decreased MD in asymptomatic carriers in subcortical structures, and increased MD in symptomatic carriers. The CTh results in our presymptomatic group, although only at trend level, also agree with previous studies reporting increases in CTh or volume in presymptomatic ADAD.^34,36,37^ Overall, our results are in agreement with previous studies that have assessed brain microstructure and macrostructure in ADAD.^34,35^ In preclinical sporadic AD, nonlinear trajectories have been described for MD and CTh.^13,15^ This nonlinear trajectory would be the result of the transition from an amyloid-negative healthy stage to an amyloid-positive preclinical and then clinical disease stage. However, data supporting nonlinear trajectories in ADAD are less clear,^34^ suggesting that participants harboring a mutation present abnormal increases in CTh from very early ages.^37^ Given these previous reports in ADAD and the small sample size of our study, we were restricted to testing linear models, but future studies with larger cohorts are warranted to test more complex nonlinear trajectories.

The main finding of our study was the significant map-to-map local association between ^11^C-DED binding and cortical microstructure and macrostructure in ADAD. This is the first time that evidence of the structural impact of brain astrocytosis as measured by ^11^C-DED binding has been reported in ADAD. Interestingly, ^11^C-DED binding had a negative association with MD, but a positive association with CTh. This finding is consistent with the divergent behavior of MD and CTh measures as previously reported in sporadic AD and in frontotemporal dementia,^15,38^ where increased CTh was related to decreased MD, while atrophy co-occurs with increased MD. The novelty of the present study is that, although MD and CTh have divergent directions, they are both topographic biomarkers whereby decreased MD and increased CTh both reflect a common underlying neuroinflammatory process as measured by ^11^C-DED binding.

Our results are biologically plausible. Neuroinflammation has been postulated as central to AD pathogenesis,^4^ and the elevated ^11^C-DED binding in presymptomatic carriers was expected, as previously published.^12^ In this presymptomatic stage, the inflammatory process would produce changes in cell phenotype including increased cell volume (neuronal or glial swelling) and cell number (glial recruitment and activation) that could explain the decrease in cortical MD.^15^ Indeed, it has been reported that changes in cell volume or glial activation can alter the microstructural properties of brain tissue.^39^ Studies conducted by us and others that have found decreases in diffusivity in presymptomatic ADAD^34,35^ have interpreted the results using the same biological rationale. Recent advances in PET technology have allowed this hypothesis, which was driven by animal studies and pathologic data, to be now tested in vivo. Consonant with our main hypothesis, our ^11^C-DED binding results favor the interpretation that astrocyte reactivity may underlie the observed local structural changes in presymptomatic carriers.

In symptomatic carriers, the observed increase in cortical MD was consistent with our previous study in the symptomatic stage of sporadic AD.^15^ In this stage, loss of tissue integrity and breakdown of cell membranes and intracellular organelles would result in water molecules moving more easily, and thus in increased diffusivity.^40^

Interestingly, not all brain areas that showed astrocytosis presented decreased cortical MD, and vice versa. Several factors might account for this mismatch. First, only clusters surviving multiple comparisons are shown. The uncorrected map-to-map correlations between structural measures and ^11^C-DED binding showed increased spatial concordance between those measures (not shown). Second, although our study shows that both phenomena co-occur, other pathophysiologic factors apart from astrocytosis, such as microgliosis,^7,41^ neuronal hypertrophy, and changes in cell membrane permeability, could account for cortical MD changes.^18,42^ In addition, in this study we only measured one marker of astrocytosis (^11^C-DED binding), and therefore we cannot exclude that other local processes measured using alternative markers of astrocytosis or microgliosis^7,41^ may contribute to MD changes in any given region.

The results of this study have several implications. First, it is evident from our diffusivity results and previous studies by us and others in patients with sporadic AD that cortical MD is decreased in early preclinical stages.^15,43^ This finding is followed by widespread increases in cortical MD once the disease advances to the symptomatic stage.^44,45^ A neuroinflammatory mechanism common to both sporadic and familial AD may underlie these similar patterns of cortical microstructural and macrostructural changes, reinforcing the role of neuroinflammation in disease pathogenesis. Second, we have shown that brain inflammation has a structural correlate and, more importantly, that these brain changes can be measured when analyzing both the diffusion properties of brain tissue and its CTh. The fact that cortical MD is related to brain astrocytosis reinforces the value of assessing the microstructural properties of the tissue, as suggested previously,^15,40^ and further research is warranted to investigate the potential utility of cortical MD as an early biomarker. A third important implication involves clinical trial design. Inflammation biomarkers are of interest in clinical trials, for patient stratification and to track biological effects of drugs.^4^ In this respect, our results using ^11^C-DED PET and recent studies emphasizing the role of astrocyte biomarkers in AD^46,47^ motivate further research on the use of astrocyte PET biomarkers in clinical trials. Our results also showed that both MD and CTh are topographic biomarkers secondary to neuroinflammatory processes as measured by ^11^C-DED PET, supporting the potential added value of MD and CTh as AD biomarkers. The fact that the CTh results were less prominent than the cortical MD results may indicate that cortical microstructure biomarkers are more sensitive than macrostructure. In this regard, we recently reported that cortical MD is more sensitive than CTh to detect neurodegenerative processes in frontotemporal dementia^38^ and sporadic AD (in preparation). In addition, this study could affect the interpretation of MRI biomarkers as surrogate markers in clinical trials. What kind of biomarker changes would be expected in trials aimed at reducing glial activation and neuroinflammation? If our interpretation is correct and brain inflammation produces cortical thickening and decreased cortical diffusivity, a drug that effectively decreases brain inflammation could produce the opposite effect: it would contribute to cortical thinning and increased diffusivity with respect to the pretreatment state. This counterintuitive effect would support the notion that brain shrinkage after immunotherapy in active (AN1792)^48^ and passive (solanezumab^49^ and bapineuzumab^50^) immunization trials was caused directly or indirectly by reducing inflammation.

The strengths of this study include its multimodal imaging approach and the accurate surface-based method, which were chosen to overcome processing limitations highlighted in the literature.^15^ More fundamentally, the unique cohort and multimodal MRI-PET data in this study provide valuable insights into the structural correlates of neuroinflammation in ADAD. The study has some limitations. First, the number of participants included is small and the results should be interpreted with caution and as a preliminary, proof-of-concept study. Nevertheless, we emphasize the rarity of the condition, the uniqueness of this sample with ^11^C-DED PET, the unicentric nature of this cohort, and that only results that survived multiple comparisons are presented. Second, diffusion MRI is particularly susceptible to field artifacts. The lack of gradient field maps did not allow us to perform a physics-based correction of echoplanar imaging distortion. Finally, although cortical MD and astrocytosis are related, given the cross-sectional design of the study, we could not infer causality with the presented data. Only longitudinal studies with long follow-up can establish the sequence of events that take place in AD.

This study shows that astrocytosis is an early event in familial AD that peaks during the presymptomatic stage. Importantly, this presymptomatic astrocytosis has a structural brain correlate that is measurable as decreased cortical MD and increased cortical thickness. Changes in brain astrocytosis, microstructure, and macrostructure occur simultaneously as the disease progresses, leading to decreased astrocyte activation in the symptomatic phase of the disease as diffusivity increases and the cortex thins. These results should be considered in clinical trials so that neuroimaging biomarkers can be interpreted correctly when used as outcome measures, and also when modeling predicted outcomes in response to treatment.

## Glossary

AD: Alzheimer disease
ADAD: autosomal-dominant Alzheimer disease
^11^C-DED: ^11^C-deuterium-L-deprenyl
CTh: cortical thickness
DTI: diffusion tensor imaging
DWI: diffusion-weighted imaging
EYO: estimated number of years to symptom onset
FWE: family-wise error
GM: gray matter
MAO-B: monoamine oxidase-B
MD: mean diffusivity
TE: echo time
TR: repetition time
